# Development of an immunoassay for the simultaneous detection of GADA and ZnT8A in autoimmune diabetes using a ZnT8/GAD65 chimeric molecule

**DOI:** 10.3389/fimmu.2023.1219857

**Published:** 2023-08-03

**Authors:** Aldana Trabucchi, Silvina Sonia Bombicino, Adriana Victoria Sabljic, Juan Ignacio Marfía, Alexandra Marisa Targovnik, Rubén Francisco Iacono, María Victoria Miranda, Silvina Noemí Valdez

**Affiliations:** ^1^ Universidad de Buenos Aires (UBA), Facultad de Farmacia y Bioquímica, Departamento de Microbiología, Inmunología, Biotecnología y Genética, Cátedra de Inmunología, Buenos Aires, Argentina; ^2^ Consejo Nacional de Investigaciones Científicas y Técnicas (CONICET) Universidad de Buenos Aires, Instituto de Estudios de la Inmunidad Humoral “Prof. Ricardo A. Margni” (IDEHU), Buenos Aires, Argentina; ^3^ Universidad de Buenos (UBA), Facultad de Farmacia y Bioquímica, Departamento de Microbiología, Inmunología, Biotecnología y Genética, Cátedra de Biotecnología, Buenos Aires, Argentina; ^4^ Consejo Nacional de Investigaciones Científicas y Técnicas (CONICET) Universidad de Buenos Aires, Instituto de Nanobiotecnología (NANOBIOTEC), Buenos Aires, Argentina

**Keywords:** autoimmune diabetes mellitus, ZnT8/GAD65 chimera, insect cells, autoantibodies, ELISA

## Abstract

**Introduction:**

The combined presence of autoantibodies to the 65 kDa isoform of glutamic acid decarboxylase (GADA) and to the islet-specific cation efflux transporter ZnT8 (ZnT8A) in serum is the best predictive sign of the loss of immune tolerance and the clinical manifestation of autoimmune diabetes mellitus (DM). The screening of GADA and ZnT8A could help to reach to a correct diagnosis and to start an early and adequate treatment. The aim of the study was to develop an immunoassay for the simultaneous detection of these autoantibodies using a chimera molecule that includes the immunodominant regions of ZnT8 and GAD65, expressed by baculovirus-insect cells system.

**Materials and Methods:**

ZnT8/GAD65 was expressed using the Bac to Bac™ baculovirus expression system. The recombinant chimera was purified by an His_6_-tag and identified by SDS-PAGE and western blot analysis, and by an indirect ELISA using specific antibodies against ZnT8 and GAD65. A fraction of ZnT8/GAD65 was biotinylated. A bridge ELISA (b-ELISA) was developed using ZnT8/GAD65 immobilized in polystyrene microplates, human sera samples from healthy individuals (n = 51) and diabetic patients (n = 49) were then incubated, and afterwards ZnT8/GAD65-biotin was added. Immune complexes were revealed with Streptavidin-Horseradish Peroxidase. Results were calculated as specific absorbance and expressed as standard deviation scores: SDs.

**Results:**

ZnT8/GAD65 was efficiently produced, yielding 30 mg/L culture medium, 80% pure. This recombinant chimera retains the immunoreactive conformation of the epitopes that are recognized by their specific antibodies, so it was used for the development of a high sensitivity (75.51%) and specificity (98.04%) b-ELISA for the detection of ZnT8A and/or GADA, in a one-step screening assay. The ROC curves demonstrated that this method had high accuracy to distinguish between samples from healthy individuals and diabetic patients (AUC = 0.9488); the cut-off value was stablished at 2 SDs.

**Conclusions:**

This immunoassay is useful either to confirm autoimmune diabetes or for detection in routine screening of individuals at risk of autoimmune DM. As DM is a slow progress disease, remaining asymptomatic for a long preclinical period, serological testing is of importance to establish a preventive treatment.

## 1 Introduction

There are several organ-specific autoimmune diseases, type 1 Diabetes Mellitus (DM1) is one of them, in which the target organ is the pancreas. This pathology is caused by the autoaggression of T lymphocytes (LT) CD8+ and CD4+ to insulin-producing pancreatic beta cells that leads to their destruction. Because of this destruction, autoantibodies against different beta cells structures are elicited, and these are the first detectable signal of an underlying autoimmune process. There are four main disease-related autoantibodies that have been shown to predict clinical DM1 (1) these are: insulin/proinsulin autoantibodies (IAA/PAA), autoantibodies to the 65 kDa isoform of glutamic acid decarboxylase (GADA), autoantibodies to the protein tyrosine phosphatase-related IA-2 molecule (IA–2A) and autoantibodies to the islet-specific cation efflux transporter ZnT8 (ZnT8A). The combined presence of these autoantibodies in serum is the best predictive sign of both the loss of immune tolerance and the clinical manifestation of the pathology.

During the progression of the disease, an inflammatory environment called insulitis is created; this is produced by the infiltration of immune cells into the pancreas. In this context, the exposure of the islet antigens of the pancreas in the HLA class I increases, triggering and accelerating the development of diabetes (2, 3).

Although up to now it has not been possible to attribute a clear role for the humoral immune response in the etiopathogenesis of autoimmune DM, the early detection of the markers IAA/PAA, GADA, IA-2A and ZnT8A makes it possible to support differential diagnosis of the several types of DM, such as Immune Mediated Autoimmune Diabetes in Adults (4, 5). A link was found between the number of positive markers in asymptomatic individuals within risk groups (direct relatives of diabetic patients), and the probability of developing the disease over a given period (6). In addition, prospective and predictive studies have shown that these markers are associated with the self-injurious cellular response of beta cells and anticipate the appearance of the first symptoms of diabetes by months or even years (7).

These autoimmune humoral markers are commonly assessed using Radioligand Binding Assay (RBA), which is considered the gold standard method (8–10). However, this approach involves the use of radioactive tracers such as [^125^I] or [^35^S]-Methionine, making it environmentally unsuitable, expensive, and restricted to authorized laboratories. To overcome the limitations of RBA and replace radiometric fluid-phase assays, alternative solid-phase tests like Enzyme-Linked Immunosorbent Assays (ELISA) have been developed in various formats (11–14). Nonetheless, ELISAs are less sensitive and specific compared to RBA, resulting in the misdiagnosis of a significant number of patients. Despite this drawback, ELISA is more commonly employed than RBA for GADA and ZnT8A determination, and as a result, numerous commercially available kits or in-house designs are used.

GADA (15) and ZnT8A (8, 16, 17) represent a high individual and combined prevalence in onset patients with DM1. The association of both markers would be an interesting rational combination for the search of autoimmunity in the infant and adolescent population, as well as in those patients in whom latent autoimmune diabetes of adults (LADA) is suspected (18). The screening of GADA and ZnT8A could be an appropriate alternative to identify diabetic subjects with underlying autoimmunity, helping to reach to a correct diagnosis and guaranteeing the start of an early and adequate treatment. This leads to the delay in the appearance of chronic complications of the disease, which strongly impacts in benefits for the patient, as well as the reduction of the socioeconomic costs associated with the pathology.

The presence of diabetes-associated autoantibodies predicts progression to future insulin dependence after diagnosis of diabetes. In this sense, according to a UKPDS (UK Prospective Diabetes Study), at least 50% of patients with autoimmune DM with onset in adulthood required insulin treatment 6 years after diagnosis (19). An important feature in this group of patients is that they have functioning beta cells at the time of diagnosis, indicating that therapeutic strategies should be implemented urgently in order to improve their metabolic control as well as to preserve insulin secretory capacity (20). It should be noted that before deciding on a therapeutic route, the estimation of C-peptide and diabetes-associated autoantibodies are highly relevant for the establishment of a personalized therapy since they are the fundamental characteristics of the disease. Likewise, the identification of an inflammatory context by determining associated cytokines and autoreactive cells against pancreatic beta cell antigens could contribute to improving this diagnosis and thus to the establishment of an adequate therapy.

In this sense, the aim of the present study is to develop a bridge ELISA (b-ELISA) for the detection of ZnT8A and/or GADA, for the diagnosis of autoimmune diabetes using a chimera molecule including immunodominant regions of the both antigens expressed by baculovirus-insect cells system. This test would be useful for screening the population at risk for DM1, as well as for the search for autoimmunity in cases of suspected LADA.

## 2 Materials and methods

### 2.1 Recombinant virus construction

The design of the chimeric molecule contained the coding sequence of the C-terminal domain of human ZnT8, which consisted of a dimer that includes amino acids 268–369 [R325] and amino acids 268–369 [W325], together with full-length human GAD65. Also, a histidine-hexapeptide (His_6_) tail at the N-terminus was added. This coding sequence was synthesized by GenScript Corporation (Piscataway, NJ, USA; www.GenScript.com). Codon optimization for insect cell expression was done.

**Figure 1:**
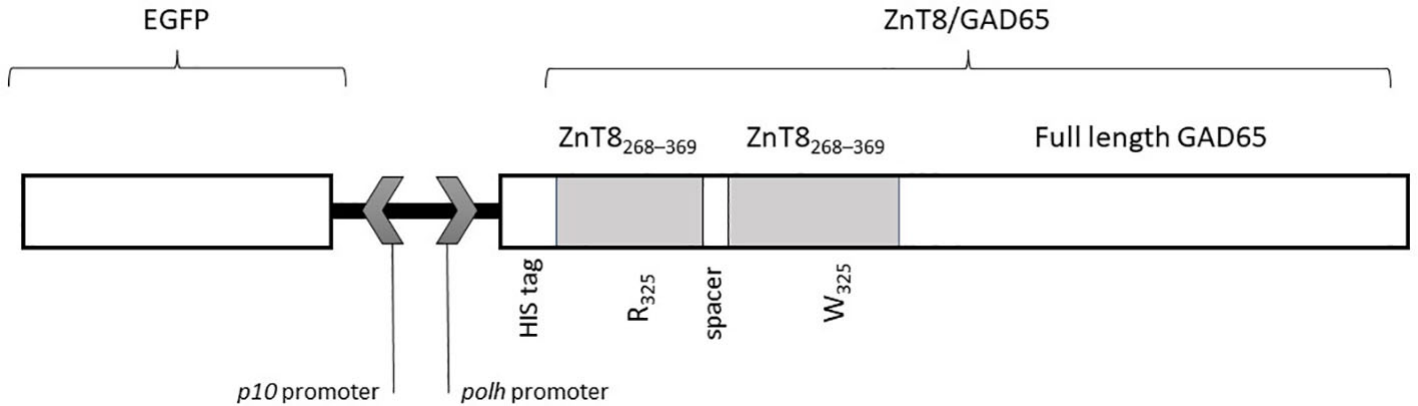
Scheme of the sequence of the recombinant baculovirus (Acpolh-ZnT8/GAD65) for the expression of ZnT8/GAD65 chimera under the polh promoter. The chimera includes two sequences of the C-terminal region of ZnT8 (residues 268-369) with arginine (R) or tryptophan (W) in the residue 325, and full length GAD65. The N-terminal includes a histidine (His_6_) tag.

The chimera cassette was directly cloned under the polyhedrin (polh) promoter using the EcoR1 and XbaI site, into the pFastBac™ Dual vector (Thermo Fisher Scientific, Waltham, MA, USA), previously modified with the enhanced green fluorescent protein (EGFP) cDNA under the p10 promoter (21). The final construction pFBD‐p10‐EGFP‐polh‐ZnT8/GAD65 is shown in **Figure 1**.

**Figure 2:**
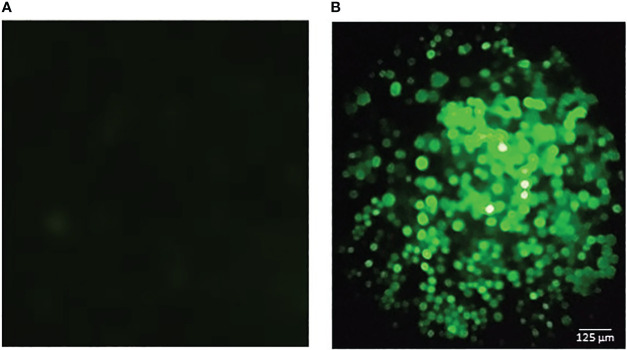
Fluorescence microscopic observation of Sf9 cells infected with the baculovirus expressing ZnT8/GAD65. The green fluorescence is due to the expression of GFP (green fluorescence protein) as an infection control. **(A)** negative control (uninfected cells). **(B)** infected cells. The bar represents 125 μm.

The recombinant baculoviruses were obtained using the Bac to Bac™ baculovirus expression system (Thermo Fisher Scientific), following the manufacturer’s instructions, as we have previously described (22). The pFBD‐p10‐EGFP‐polh‐ZnT8/GAD65 vector was transformed into a chemically competent Escherichia coli DH10Bac™ strain (Thermo Fisher Scientific) by heat shock to generate the recombinant bacmid by transposition. Then, the bacmids were purified, the sequences were verified by Sanger sequencing (GenBiotech, Buenos Aires, Argentina) and finally used to transfect one million Sf9 cells by using Cellfectin II Reagent (Thermo Fisher Scientific). After 4‐days incubation at 27°C, the cell culture supernatant was collected and centrifuged at 500 x g for 10 min. The transfection efficiency was determined by measuring the EGFP expression by fluorescence under UV light (**Figure 2**). The recombinant Autographa californica nuclear polyhedrosis virus was named Acpolh‐ZnT8/GAD65.

Then, a round of amplification was performed in Sf9 cells seeded in T‐25 flasks at 27°C, at a multiplicity of infection (MOI) of 0.02.

The Sf900 II insect cell culture medium was from Invitrogen™ (Gaithersburg, MD, USA) and the antibiotic and antimycotic solutions (Anti-Anti 100X) and the fetal bovine serum were from GIBCO (Thermo Fisher Scientific). The amplified Acpolh‐ZnT8/GAD65 was titrated by plaque assay (23). This high‐titer Acpolh‐ZnT8/GAD65 was the viral stock used for protein production in insect cell.

### 2.2 Recombinant ZnT8/GAD65 expression and purification from cell cultures

For the ZnT8/GAD65 expression, independent Sf9 (serial passage 30) suspension cultures in log-phase at a cell density of 4 x 10^7^ cells in 30 mL (2 x 10^6^ cell/mL) were infected with Acpolh‐ZnT8/GAD65 at MOI 1. After 4 days of incubation in the dark at 27°C, in continuous agitation, cells were centrifuged. The recombinant chimera was recovered following the protocols of lysis and purification as previously described (24).

For the determination of total protein concentration Bradford microassay (25) was performed using Coomassie Plus™ Protein Assay (Thermo Fisher Scientific).

The recombinant ZnT8/GAD65 chimera was stored with a mixture of 50% v/v glycerol, Tween 20 and protease inhibitors, and kept at -20°C until used.

### 2.3 Sodium dodecyl sulphate-polyacrylamide gel electrophoresis and western blot analysis

The protocols followed for Sodium dodecyl sulphate-polyacrylamide gel electrophoresis (SDS-PAGE) and Western Blot (WB) analysis were the same as Faccinetti et al. (26) and Trabucchi et al. (24). Briefly, fractions at different steps of purification of ZnT8/GAD65 were collected, diluted in 0.2 mL of SDS-PAGE sample buffer (50 mM Tris–HCl, 12.0% glycerol, 0.005% bromophenol blue, 4.0% SDS, 4.0% 2ME, pH 6.8), boiled for 5 minutes and separated by 10% SDS-PAGE followed by Coomassie Brilliant Blue R-250 staining. For detection by WB, once the protein bands were transferred onto nitrocellulose membranes, the expression of the chimera was evidenced by either a mouse monoclonal antibody to GAD65 1/200, a rabbit polyclonal antibody to ZnT8 1/200 or a mouse monoclonal anti-histidine antibody 1/3000 (BD Pharmingen, San Diego, CA, USA). Bound antibodies were visualized by incubation with peroxidase-conjugated goat antibodies to mouse IgG 1/2000 or with peroxidase-conjugated goat antibodies to rabbit IgG 1/2000, respectively. Protein bands were visualized by the addition of α-chloronaphthol (Sigma-Aldrich Inc., St Louis, MO, USA) and 10 vol. H_2_O_2_.

### 2.4 Identification of the recombinant ZnT8/GAD65 protein expressed in Sf9 cells by indirect ELISA

The identification of purified chimera was evaluated by means of indirect enzyme linked immunosorbent assay (ELISA) using mouse monoclonal antibodies against GAD65 and a rabbit serum against ZnT8. For this purpose, polystyrene microplates (Maxisorp, NUNC, Roskilde, Denmark) were coated overnight (ON) at 4°C with 0,1 ug of purified ZnT8/GAD65 chimera per well, washed three times with phosphate buffered saline (PBS: 1.5 mM KH_2_PO_4_, 8.1 mM Na_2_HPO_4_, 140 mM NaCl, 2.7 mM KCl, pH 7.4), blocked for 1.5 h with 200 μL of blocking solution (3% skim milk in PBS) per well, and washed five times with PBS containing 0.05% Tween 20 (PBS-T). Samples diluted 1/100 in 3% skim milk in PBS-T (PBS-MT) were evaluated five times, and microplates were incubated for 1 h. After another round of 5 washes with PBS-T the bound specific antibodies were detected by the addition of peroxidase-conjugated goat antibodies to mouse IgG or with peroxidase-conjugated goat antibodies to rabbit IgG (Jackson ImmunoResearch Laboratories, Inc., West Grove, PA, USA) diluted 1/3000 in PBS-MT. Following washing (five times with PBS-T plus one final wash with PBS), the chromogenic substrate was added (3,3′,5,5′-tetramethyl-benzidine/H_2_O_2_ mixture; Single Component TMB Peroxidase EIA Substrate Kit, BioRad, Hercules, CA, USA), and plates were incubated for 15 min in the dark. The color reaction was stopped with 4N H_2_SO_4_. The oxidized substrate was measured at 450 nm with an ELISA plate reader MultiskanFC (Thermo Scientific Labsystems). The blank control was made by replacing serum samples with PBS‐MT. Results were expressed as specific absorbance (A = the mean absorbance of each sample minus the mean absorbance of the blank control).

### 2.5 ZnT8/GAD65 application in immunoassay for the simultaneous detection of ZnT8A and GADA

#### 2.5.1 Sera collection

Sera from diabetic patients (n = 49) were selected among the samples collected in our laboratory during the routine detection of autoantibodies (Servicios Tecnológicos de Alto Nivel, STAN-CONICET).

Control sera were obtained from 51 healthy subjects without personal or family history of autoimmune disease.

This work was performed with the approval of the Ethical Committee of José de San Martín Clinical Hospital, Buenos Aires, Argentina.

All experiments were done in accordance with the relevant guidelines and regulations.

Written informed consent was obtained from all participants.

#### 2.5.2 Biotinylation of the chimera ZnT8/GAD65

Biotinylation of the purified chimera was done as we have previously described (24, 27). Two hundred eighty micrograms (280 ug) of desalted protein were incubated with excess of sulfo-NHS-biotin (Pierce Biotechnology, Rockford, IL, USA), and free biotin was then removed.

ZnT8/GAD65-biotin was stored until use at -20°C with the addition of 50% v/v glycerol, Tween 20 and protease inhibitors.

#### 2.5.3 Bridge ELISA

The protocol employed was based on that previously described (14, 24, 27), with minor modifications. Briefly, polystyrene microplates were coated ON at 4°C with 0.1 ug of purified ZnT8/GAD65 chimera per well. After washing with PBS, adding 200 μL of blocking solution per well for 1.5 h, and washing five times with PBS-T, 50 ul samples were added in duplicate. After 1 h of incubation at room temperature, plates were washed again and 56.0 ng of ZnT8/GAD65-biotin per well were added. One hour later, another round of washing steps was done, and the bound ZnT8/GAD65-biotin was detected by the addition of Streptavidin-Horseradish Peroxidase (Jackson ImmunoResearch Laboratories, Inc.). Finally, microplates were washed five times with PBS-T plus one final washing step with PBS. The color reaction was elicited by the addition of the chromogenic substrate incubated for 15 minutes in the dark and was then stopped with 4N H_2_SO_4_. The oxidized substrate was measured at 450 nm with an ELISA plate reader. The blank control was made by replacing serum samples with PBS‐MT. Results were calculated as specific absorbance and expressed as standard deviation scores: SDs = (A‐Ac)/SDc, where Ac is the mean specific absorbance of 25 healthy control sera and SDc its standard deviation. The cut‐off value of the assay was set at 2 SDs.

### 2.6 Statistical analysis

The normal distribution of the data was analyzed by the D’Agostino & Pearson Omnibus normality test. To select the cut-off with maximum sensitivity and specificity, ROC curves were made by plotting these parameters against the corresponding cut-off values. Statistical significance was assessed by parametric tests: Student’s t-test for unpaired samples with Welch correction; or non-parametric tests: Mann-Whitney U test for unpaired data, when applicable. Calculations were performed using GraphPad Prism version 6.01 for Windows (GraphPad Software, San Diego California, USA, www.graphpad.com). A value of p <0.05 was considered statistically significant.

## 3 Results

### 3.1 Generation of the recombinant baculovirus Acpolh-ZnT8/GAD65

For the construction of the chimera, we based ourselves in the knowledge of the immunodominant regions of both proteins. As previously described by Rickert et al. (28), GAD65 has nine conformational epitopes throughout its entire structure so we decided to use full length GAD65. As for ZnT8, it is known that C-terminal domain (amino acids 268–369) is the region most recognized by specific autoantibodies (8). Also, this region contains a polymorphism in amino acid 325. In this sense, we decided to use a heterodimeric variant of C-terminal domain containing R or W in 325 position, based on that we have previously demonstrated that this heterodimeric construction showed higher signal levels and increased dynamic range in RBA for ZnT8A detection (29).

The cassette containing a dimer of ZnT8 and full length GAD65, fused to a His_6_-tag was cloned under the control of the polyhedrin promoter, hence pFBD-polh-ZnT8/GAD65 was obtained. The N-terminal His_6_-tag was added to facilitate the purification of the recombinant chimera by Ni^2+^–NTA Immobilize Metal Affinity Chromatography. The expression vector used in this work has another distinctive feature, which is the employment of EGFP, cloned under the p10 promoter, as a reporter gene useful for visualizing viral infection (**Figure 2**). The corresponding bacmids were produced with these plasmids. The Acpolh-ZnT8/GAD65 virus was finally obtained by transfection of the bacmids and amplification in Sf9 cells.

### 3.2 Recombinant ZnT8/GAD65 expression and purification from cell cultures

**Figure 3:**
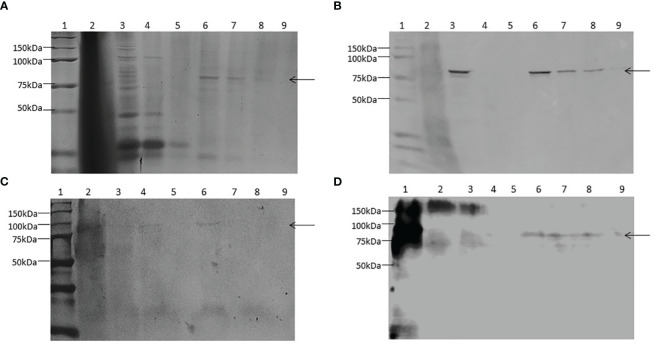
Expression and purification of ZnT8/GAD65 in Sf9-cells. **(A)** SDS-PAGE (10.0% T 6.0% C, 1.5 mm, under reducing conditions, stained with Coomassie Brilliant Blue R-250); **(B)** WB revealed with monoclonal antibodies to GAD65 as primary antibody; **(C)** WB revealed with polyclonal antibodies to ZnT8 as primary antibody; **(D)** WB revealed with monoclonal antibodies to His_6_ as primary antibody. Samples: 1. Molecular weight markers, 2. Pellet, 3. Total soluble fraction, 4. Unbound material, 5. Wash step, 6-9. Consecutive eluates of purified ZnT8/GAD65. Arrows indicate the electrophoretic mobility of ZnT8/GAD65.

The recombinant chimera was expressed in Sf9 cells with a MOI of 1 based on previous experiences (24). After 4 days of infection, we efficiently produced ZnT8/GAD65, with a yield of 30 mg of chimera per litre of culture medium, 80% pure. SDS-PAGE analyses of total cell lysates and the different purification steps showed one band of approximately 80 kDa, the expected molecular weight for the full-length engineered chimera (**Figure 3A**). Moreover, the analysis by WB allowed identifying the recombinant chimera using specific antibodies to GAD65 (**Figure 3B**), to ZnT8 (**Figure 3C**) or to His_6_ (**Figure 3D**).

### 3.3 Identification of the recombinant ZnT8/GAD65 protein expressed in Sf9 cells by indirect ELISA

**Figure 4:**
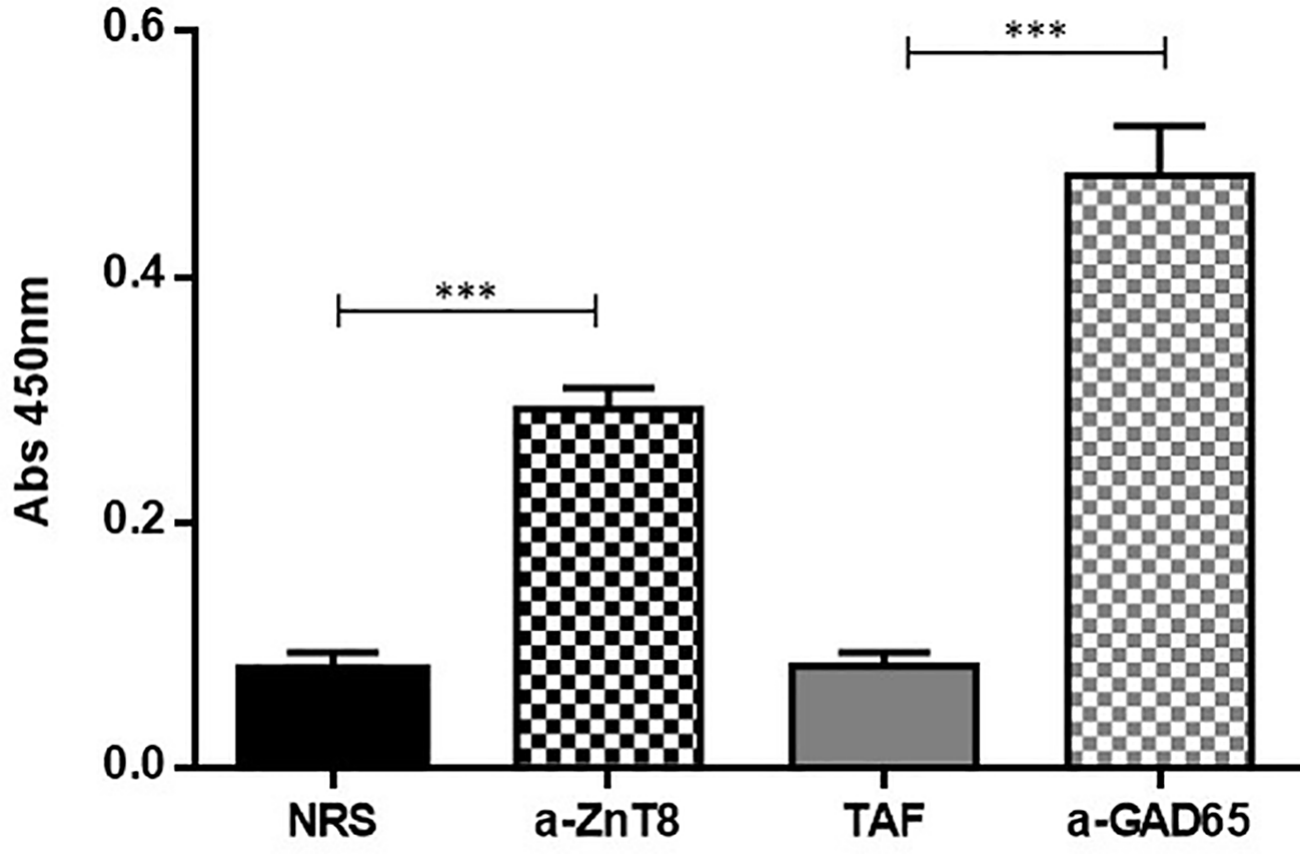
Indirect ELISA for the characterization of the recombinant ZnT8/GAD65 chimera. NRS, Normal Rabbit Serum used as control; a-ZnT8, hyperimmune rabbit serum against human ZnT8; TAF, Total Ascitic Fluid from mouse used as control; a-GAD65, monoclonal mouse antibodies against human GAD65. (***p < 0.0001).

Identification of the ZnT8 and GAD65 domains in the recombinant chimera was evaluated by indirect ELISA using ZnT8/GAD65 as the immobilized antigen that interacted with polyclonal rabbit ZnT8 antibodies and monoclonal mouse GAD65 antibodies. As it is shown in **Figure 4**, both domains were correctly recognized by their specific antibodies, demonstrated by the significant difference of the signals obtained with the specific antibodies with respect to the normal controls (p < 0.0001).

### 3.4 ZnT8/GAD65 application in immunoassay for the simultaneous detection of ZnT8A and GADA

The assay developed herein is based on the double interaction of specific antibodies with the chimeric molecule. On the one hand, ZnT8/GAD65 is immobilized on the plastic surface of the well (solid phase), which interacts with one paratope of the antibody, leaving the other paratope free to interact with the biotin-labelled chimera (ZnT8/GAD65-biotin) in a fluid phase.

**Figure 5:**
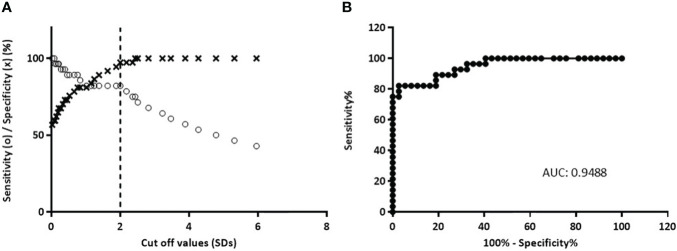
Analysis of b-ELISA performance resulting from the study of 51 sera from normal control individuals and 49 sera from diabetic patients. **(A)** Sensitivity curve (o) and specificity (×) as a function of the possible cut‐off values. The vertical dashed line indicates the cut‐off value with the optimized sensitivity and specificity parameters (cut‐off = 2.0). **(B)** ROC curve analysis of b-ELISA, AUC is included.

**Figure 6:**
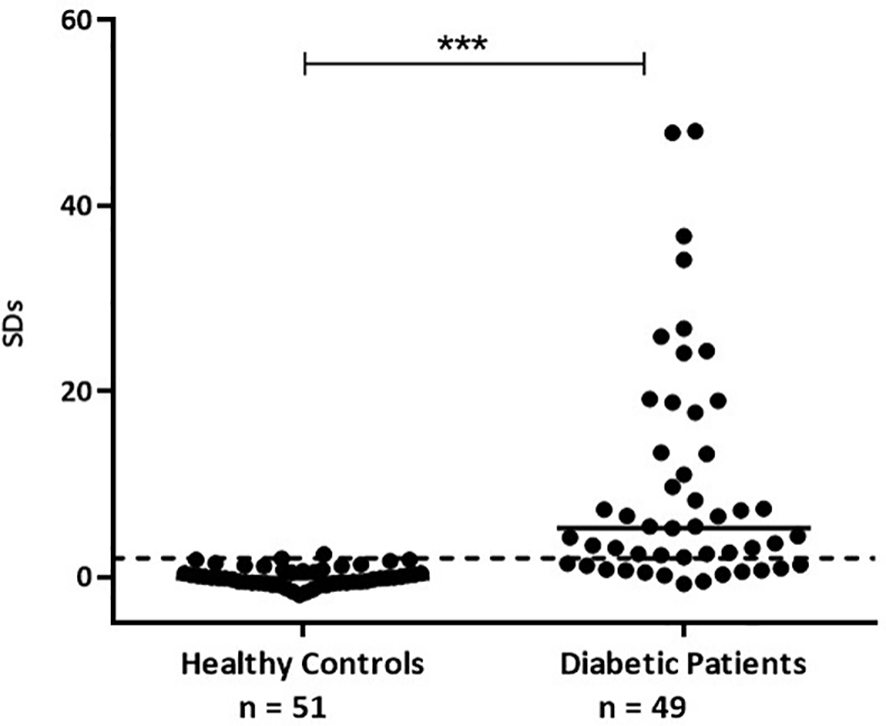
GADA and ZnT8A results obtained by b-ELISA in sera from healthy control individuals and sera from diabetic patients. Results are expressed as SDs. The cut‐off value (SDs > 2.0) is indicated by a dotted line and medians for each population are indicated as a full line (***p < 0.0001).

For the optimization of this immunoassay, we selected 49 sera samples from diabetic patients collected in our laboratory during the routine detection of humoral markers, together with 51 sera samples from healthy control individuals without history of autoimmune diseases. This assay presented a wide dynamic range (-2.00 to 48.00 SDs). The specificity was 98.04%, this is 100% minus the percentage of false positives calculated with healthy control individuals. By means of receiver operating characteristic curves (ROC) where the effect of different cut-off values was evaluated in terms of specificity and sensitivity, the performance of the test was optimized (**Figure 5**). This method had high accuracy to distinguish between samples from healthy individuals and diabetic patients as shown by the area under the ROC curve (AUC = 0.9488) (30). Accordingly, the cut-off value was stablished at 2 SDs. Thus, 37 (75.51%) out of the 49 patients’ sera analyzed, were detected as positive for ZnT8A and/or GADA, with SDs ranging from −0.814 to 47.99 and a median of 5.222 (**Figure 6**). To calculate the coefficient variation, a positive GADA and ZnT8A serum from a diabetic patient was employed. The intra‐assay coefficient variation was 6% and the inter‐assay coefficient variation was 4.7%.

## 4 Discussion

At present, DM is a significant social health concern due to its prevalence, specific complications, and its association with other pathologies. Autoimmune DM, a relatively common disorder, occurs in individuals with genetic susceptibility and is linked with anti-islet autoantibodies that can be present for years before the onset of clinical symptoms. The detection of these autoantibodies is crucial for predicting and diagnosing autoimmune diabetes, and it is also important for the design of disease prevention trials. A range of well-characterized assays (RBA, ELISA, electrochemiluminescence assays) is available for identifying these autoantibodies, which undergo evaluation through international quality control workshops (31, 32).

GAD65 and ZnT8 are the major autoantigens in autoimmune DM. As this pathology has a long asymptomatic preclinical period where the autoimmune process is silent, the detection of the related autoantibodies (GADA and/or ZnT8A) in diabetic patients and the individuals at risk, is the first evidence that the pancreatic beta cells are being destroyed. That is why both autoantibodies became a promising tool as humoral immune markers for the early diagnosis of autoimmune DM.

Among the different clinical phenotypes associated with autoimmunity in diabetes, we can encounter patients with DM1 with an abrupt onset, where multiple antibodies typically appear. On the other hand, there are patients with slowly progressive autoimmune forms of diabetes (Adult-Onset Autoimmune Diabetes), in which the most frequent antibodies are GADA and/or ZnT8A. Regardless of the severity of the clinical presentation, the developed analytical tool would enable the detection of the associated autoimmune component.

As it was mentioned before, GADA and ZnT8A are one of the earliest detectable islet cell autoantibodies, and are considered highly predictive for the development of autoimmune DM. Therefore, the serological testing of these antibodies is extremely important to establish an adequate preventive treatment, which allows avoiding an abrupt onset of ketoacidosis and a strong metabolic imbalance. RBA is the reference method for the detection of both markers, yet this method is costly, and environmentally inappropriate due to the use of radioactive materials. So, it is necessary to have low-cost immunoanalytical strategies that are feasible to be carried out in laboratories of medium and low complexity.

In autoimmune DM, the autoantibodies involved mainly recognize discontinuous conformational epitopes of the autoantigens. For this reason, in the development of immunoanalytical methodologies for the detection of these autoantibodies, it is necessary to have a recombinant antigen production system that guarantees both correct folding and high expression yield. In this sense and given that the proposed chimera constitutes a high molecular weight protein, difficult to be expressed with correct folding in *E. coli*, the eukaryotic expression system such as baculovirus-insect cells represents an interesting strategy for the achievement of our aim. ZnT8 and GAD65 could be successfully combined into a single molecule where each antigen retained its immunoreactivity.

The cells infected with the recombinant baculovirus Acpolh-ZnT8/GAD65 expressed, in addition to the chimera molecule of interest, the EGFP protein, which allowed us to visualize the viral infection by observation under UV light. After 4 days of infection, it was possible to obtain ZnT8/GAD65 80% pure with a yield of 30 mg/L of culture medium, revealed by SDS-PAGE and WB analysis. Even though the purity of the expressed protein was not extremely high, it was suitable for our intended use, the development of highly specific and sensitive immunoassays for the detection of autoantibodies. Before setting up the b-ELISA, we evaluated the chimeric molecule by means of an indirect ELISA using anti-ZnT8 and anti-GAD65 antibodies to confirm the interaction of the epitopes of each antigen with their specific antibodies. The results obtained demonstrated that the ZnT8/GAD65 chimera preserves its immunoreactive epitopes (**Figure 4**), thus this encouraged us to go ahead with the design of a bridge ELISA for the detection of autoantibodies in individuals at risk or diabetic patients with doubtful classification.

To achieve our goal, we selected 49 serum samples from diabetic patients that were collected during routine detection of humoral markers in our laboratory. The obtained results showed a sensitivity of 75.51% with a broad range of SD signals (from −0.814 to 47.99) and a median of 5.222. These signals were significantly different from those obtained from healthy control individuals (with SDs ranging from -1,979 to 2,411 and a median of -0.1650) (see **Figure 6**), with 98.04% of specificity. Moreover, as it was demonstrated by ROC curves and AUC, when stablishing a cut-off value of 2 SDs, the assay had high accuracy to distinguish between samples from both groups under study (**Figure 5**).

Taken together, the results showed herein demonstrate that it was possible to express a chimera molecule containing the immunodominant regions of the two major DM autoantigens by a simple and low-cost expression system, with high yield and purity. This recombinant chimera retains the immunoreactive conformation of the epitopes that are recognized by their specific antibodies, so it can be use in immunoassays for the simultaneous detection of highly prevalence ZnT8A and GADA. This immunoassay is useful either to confirm autoimmune diabetes or for detection in routine screening of individuals at risk of autoimmune DM.

In this sense, since the FDA has recently approved a monoclonal antibody therapy to delay DM debut (Teplizumab), there is a need for development of low complexity methods for prediction in order to establish prevention protocols. Herein we describe the development of a screening tool appropriate for autoantibodies detection in risk groups. This is a single assay with high prevalence of detection, then if it is necessary, GADA or ZnT8A can be discriminated by a confirmation assay.

Furthermore, it is important to note that cellular auto-aggression is the cause of the pathology, therefore its study is of the utmost importance for understanding the underlying autoimmune process that leads to the onset of the disease and this knowledge it will be helpful in the development of tolerance induction strategies. Regarding this, it is our intention to use ZnT8/GAD65 as antigen in the evaluation of the cellular immune response in diabetic patients or individuals at risk, and to characterize the associated inflammatory context.

## 5 Conclusion

In this study, we describe the development of an easy-to-perform b-ELISA for the detection of autoantibodies to ZnT8 and GAD65. The assay was based on the use of a chimeric molecule that has the immunodominant epitopes of both antigens. It was highly sensitive and specific, and it was able to detect autoantibodies in patients with autoimmune DM.

The use of a chimeric molecule in the b-ELISA has several advantages over the use of individual antigens. These advantages include: (i) reduced production and purification costs, (ii) increased likelihood of exposing all epitopes involved in the interaction with autoantibodies, (iii) reduced number of necessary reagents, (iv) easier test automation and (v) simplicity in immunoassay optimization.

The b-ELISA was designed as a screening strategy for DM1. This assay is applicable to the general population, where the prevalence of DM1 is less than 0.1% (33). In this sense, it is important to highlight that in DM the highest percentage of patients with DM1 comes from the general population and not from risk groups. Therefore, having this screening assay is an interesting cost-effective option in our environment, leading to improved diagnostic accuracy and potentially more effective therapeutic interventions.

## Data availability statement

The original contributions presented in the study are included in the article/supplementary material. Further inquiries can be directed to the corresponding author.

## Author contributions

AT, SB and SV conceived and designed the experiments. AT and SB performed the experiments. AT, SB and SV analyzed and interpreted the data. AT, SV, AMT and MM contributed reagents/materials/analysis tools. AT, SB and SV wrote the paper. JM, AS and AMT contributed with the performance of experiments. MM and SV supervised the research. All authors contributed to the article and approved the submitted version.

## Glossary

**Table:**

AUC	Area under the curve
DM	Diabetes Mellitus
DM1	type 1 Diabetes Mellitus
EGFP	enhanced green fluorescent protein
ELISA	enzyme linked immunosorbent assay
GADA	autoantibodies to the 65 kD isoform of glutamic acid decarboxylase
GAD65	65 kD isoform of glutamic acid decarboxylase
IA-2A	autoantibodies to the protein tyrosine phosphatase-related IA-2 molecule
IAA/PAA	insulin/proinsulin autoantibodies
LADA	latent autoimmune diabetes of adults
LT	T Lymphocytes
MOI	multiplicity of infection
ON	Overnight
PBS	phosphate buffered saline
PBS-T	PBS with Tween 20 0.05% v/v
PBS-MT	PBS-T with 3% skim milk
RBA	Radioligand Binding Assay
ROC	receiver operating characteristic
SDs	standard deviation scores
WB	western blot
ZnT8	islet-specific cation efflux transporter ZnT8
ZnT8A	autoantibodies to the islet-specific cation efflux transporter ZnT8
